# C1q is elevated during chronic *Staphylococcus epidermidis* central nervous system catheter infection

**DOI:** 10.3389/fimmu.2024.1342467

**Published:** 2024-05-31

**Authors:** Matthew Beaver, Lara Bergdolt, Anna Dunaevsky, Tammy Kielian, Gwenn L. Skar

**Affiliations:** ^1^ Department of Pediatrics, University of Nebraska Medical Center, Omaha, NE, United States; ^2^ Department of Neurological Sciences, University of Nebraska Medical Center, Omaha, NE, United States; ^3^ Department of Pathology and Microbiology, University of Nebraska Medical Center, Omaha, NE, United States

**Keywords:** central nervous system infection, cerebrospinal fluid shunt, C1q, *Staphylococcus epidermidis*, complement

## Abstract

**Introduction:**

Significant neurologic morbidity is caused by pediatric cerebrospinal fluid (CSF) shunt infections. The underlying mechanisms leading to impaired school performance and increased risk of seizures are unknown, however, a better understanding of these mechanisms may allow us to temper their consequences. Recent evidence has demonstrated important roles for complement proteins in neurodevelopment and neuroinflammation.

**Methods:**

We examined complement activation throughout *Staphylococcus epidermidis* (*S. epidermidis*) central nervous system (CNS) catheter infection. In addition, based on accumulating evidence that C3 plays a role in synaptic pruning in other neuroinflammatory states we determined if C3 and downstream C5 led to alterations in synaptic protein levels. Using our murine model of *S. epidermidis* catheter infection we quantified levels of the complement components C1q, Factor B, MASP2, C3, and C5 over the course of infection along with bacterial burdens.

**Results:**

We found that MASP2 predominated early in catheter infection, but that Factor B was elevated at intermediate time points. Unexpectedly C1q was elevated at late timepoints when bacterial burdens were low or undetectable. Based on these findings and the wealth of information regarding the emerging roles of C1q in the CNS, this suggests functions beyond pathogen elimination during *S. epidermidis* CNS catheter infection. To identify if C3 impacted synaptic protein levels we performed synaptosome isolation and quantified levels of VGLUT1 and PSD95 as well as pre-, post- and total synaptic puncta in cortical layer V of C3 knockout (KO) and wild type mice. We also used C5 KO and wild type mice to determine if there was any difference in pre-, post- and total synaptic puncta.

**Discussion:**

Neither C3 nor C5 impacted synaptic protein abundance. These findings suggest that chronic elevations in C1q in the brain that persist once CNS catheter infection has resolved may be modulating disease sequalae.

## Introduction

Cerebrospinal fluid (CSF) shunt placement is the most common pediatric neurosurgical procedure, with tens of thousands of CSF shunts placed annually in the United States to treat hydrocephalus (1, 2). While these shunts are a life-saving intervention, they are frequently complicated by infection in 5–30% of patients (3, 4). Two-thirds of shunt infections are caused by the gram-positive pathogen *Staphylococcus epidermidis* (*S. epidermidis*), which is capable of forming a biofilm. Biofilms are communities of bacteria encased in a protective matrix, where complex structure and metabolic dormancy make treatment difficult (5–7). Due to the biofilm nature of infection, treatment of shunt infection is onerous, requiring surgical removal and placement of a new shunt following treatment with several weeks of intravenous antibiotics (8). Additionally, the host immune response to these biofilm infections is unique as they avoid immune clearance by altering the typical pro-inflammatory bactericidal host response to an anti-inflammatory one (9, 10).

Beyond these difficulties, shunt infections are associated with significant long-term neurologic sequelae. Studies have demonstrated that children with a history of shunt infection have reduced verbal and performance IQ as well as lower intelligence and significantly impaired school performance compared to their peers without infection (11–13). Shunt infection also increases seizure risk, which has been reported to be as high as 47% (5, 14, 15). The mechanisms responsible for the severe neurologic damage associated with shunt infection are unknown. A better understanding of the key mechanisms that drive chronic neurological sequelae following CSF shunt infections may allow for their targeting to mitigate adverse outcomes.

The complement system is activated during CNS catheter infection, which includes proteins that opsonize pathogens and induce inflammatory responses that assist in combating infection. Despite the role of complement in infection containment, it may exert untoward negative neurologic consequences for patients. Previous work from our laboratory has demonstrated elevated levels of complement proteins in the CSF of rats with CNS catheter infection, especially at later time points when bacterial burdens are minimal, suggesting potential activity beyond pathogen response (16). While traditionally thought of as an immune privileged environment, recent evidence has established critical roles for the complement pathway in the CNS. This includes developmentally appropriate synaptic pruning as well as in CNS inflammatory disorders. Complement proteins C1q and C3 have been implicated in microglial-mediated synaptic pruning in the mouse retinogeniculate system (17). Stevens et al. demonstrated that C1q co-localized with pre- and post-synaptic puncta that were missing a synaptic partner and that C1q knockout (KO) mice had defects in eye-specific segregation of retinal ganglion cells (17). Also, it was shown that C3 was associated with synaptic puncta and C3 KO mice recapitulated the phenotype of C1q KO animals (17).

Complement activation and pathological synaptic pruning have also been described in several neurodegenerative conditions, such as Alzheimer’s disease, multiple sclerosis, traumatic brain injury, and stroke (18–20). For example, complement has long been associated with multiple sclerosis pathology, with early animal studies of experimental autoimmune encephalitis (EAE) demonstrating reduced disease severity in animals treated with the complement inhibitor cobra venom factor (CVF) (19, 21–23). Reports in ischemic stroke have shown increased expression of C3a and C5a (24, 25). Injection of HIV Tat protein into the mouse cortex caused increased concentrations of C1q and C3 at the injection site compared to controls; however, the resulting synaptic loss was not C1q-dependent (26). C3 and Factor B are elevated in the CSF of patients with bacterial meningitis compared to aseptic meningitis (27–29). One study has reported elevated levels of soluble membrane attack complex (sMAC) in the CSF of patients with shunt infection (30).

However, roles for complement extending beyond bacterial clearance during CSF shunt infection have never been explored. Previous studies from our laboratory have demonstrated elevated levels of several complement components in the CSF at chronic intervals in a rat model of CNS catheter infection (16). Levels of some complement components peaked at time points when bacterial burdens were declining, supporting a potential longer-term role for complement in post-infectious pathology (16). Due to the wealth of literature supporting complement-mediated synaptic pruning in brain development and neuroinflammatory states, we were interested in the possibility of complement inducing synaptic loss during CSF shunt infection. The objective of this study was to examine complement activation throughout the course of *S. epidermidis* CNS catheter infection and determine if C3 or C5 leads to alterations in synaptic protein levels.

## Materials and methods

### Animals

All *in vivo* experiments were performed using equal numbers of male and female, 8- to 9-week-old, corresponding to adolescents, C3 knockout (KO) (RRID: IMSR_JAX:029661) mice with C57BL/6J (RRID: IMSR_JAX:000664) as controls, and C5 KO mice (RRID: IMSR_JAX:000461) with C57BL/10SnJ (RRID: IMSR_JAX:000666) as controls (The Jackson Laboratory, Bar Harbor, ME). All experimental personnel were blinded to animal species and sterile versus infected status until unblinding for data analysis. The protocol for animal use was approved by the University of Nebraska Medical Center Institutional Animal Care and Use Committee (protocol # 19–029-05-FC) and is compliant with the National Institute of Health (NIH) guidelines for the use of rodents.

### Bacterial Strain and *in vitro* propagation

*S. epidermidis* 1457 from the University of Nebraska Medical Center clinical microbiology laboratory was kindly provided by Dr. Paul Fey. This isolate was recovered from an infected central venous catheter and initially characterized by Mack et al. and has not been laboratory adapted or modified since its initial characterization (31).

### Catheter preparation and implantation into the brain lateral ventricle

Hollow silicone catheters (3 French, 1mm length, 1mm diameter; Access technologies/Norfolk Medical, Skokie, IL) were incubated overnight with *S. epidermidis* to ensure bacterial adherence to the catheter and prevent bacterial efflux upon insertion of the catheter into the lateral ventricle of the brain as previously described (32, 33). Briefly, mice were anesthetized with intraperitoneal ketamine (100 to 200 mg/kg) and xylazine (5 to 13 mg/kg) and received subcutaneous injections of bupivacaine (1 mg/kg) and buprenorphine (0.01 mg/kg) to mitigate any potential pain. Each animal was then positioned in a stereotaxic apparatus and either sterile or *S. epidermidis* catheters were placed vertically into the left ventricle via a burr hole in the skull, as previously described (32). To secure the catheter in place and reduce bacterial efflux and bleeding, bone wax (World Precision Instruments, Sarasota, FL)was used to seal the burr hole and Vetbond surgical glue to close the incision (3M, St. Paul, MN).

### Bacterial enumeration from catheters and brain parenchyma

Mice were euthanized with an overdose of inhaled isoflurane. Then catheters and associated brain tissue were collected at the designated time points post-surgery as previously described (32, 34). Briefly, catheters were rinsed in sterile phosphate-buffered saline (PBS) after removal to eliminate non-adherent bacteria and the catheter-associated biofilms were vortexed (Eppendorf MixMate) at 2000rpm for 5 min in 500 μL PBS. Tissue within 1 mm of initial catheter placement was homogenized in 500 μL sterile PBS, supplemented with a complete protease inhibitor cocktail tablet (Roche, Basel, Switzerland) and RNase inhibitor (Promega, Madison, WI), using a Polytron homogenizer (Brinkmann Instruments, Westbury, NY). A 100 μL aliquot of the brain homogenates and supernatants from vortexed catheters were used to quantify bacterial titers via tenfold serial dilutions on blood agar plates. Homogenates were also used to quantify complement component levels after centrifugation and storage at −80°C as previously described (32–34).

### Complement component analysis

C1q, Factor B, MASP2, C3, and C5 were measured by enzyme-linked immunosorbent assay (ELISA) according to the manufacturer’s specifications (LSBio, Shirley, MA) at days 1, 3, 5, 7, 10, 14, 28 and 56 post-surgery in C57BL/6J mice implanted with either sterile or *S. epidermidis* infected catheters. Fourteen to 25 mice per timepoint were used. Protein concentrations from brain samples were measured using a bicinchoninic assay (BCA; 23225 Thermo Fisher Scientific Inc., Rockford, IL) and used to normalize complement component levels to correct for any variation in tissue sample size as bacterial presence contributes a negligible amount to overall protein concentration in *S. epidermidis* infected samples.

### Synaptosome isolation

At day 28 post-infection mice (n=8–9) were euthanized with an overdose of inhaled isoflurane. Mice were then perfused with 30 ml of PBS. Brains were then removed and bisected sagittally. The hippocampus and cortex from the ipsilateral hemisphere of catheter placement were dissected and placed in ice cold 1X HBSS. Once all tissue was collected, 1 ml of homogenization buffer (Sucrose Fisher BP220–1, EDTA RPI E14000–500, Tris (Invitrogen 15504–020) with complete protease inhibitor tablets (Roche 11697498001) was added to a glass Dounce tissue homogenizer with pestle B (Kimble 885300). Brain tissue was homogenized with 10 strokes. Brain homogenates were centrifuged at 1000 X g for 10 min and the supernatant was collected. Homogenization buffer and DTT (Fisher 15–508-013) were mixed with Percoll (GE 17–0891-01) to generate solutions of 3%, 10%, 23% Percoll. Three milliliters of the 23% Percoll solution was added to an ultracentrifuge tube (Beckman Coulter 344059) and underlaid with 3 ml of the 10% Percoll solution using a borosilicate glass Pasteur pipet (Fisher 13–678-20C). Next the 3% Percoll solution was added to the supernatant to reach a final volume of 6 ml. Then the supernatant and 3% Percoll solution was overlaid on the previously made Percoll gradient. The samples were centrifuged in an SW41 rotor at 18,200 rpm for 12 min with acceleration of 8 and deceleration of 6 (Beckman Cuoulter Optima L-90K Ultracentrifuge). The band at the interface of the 23% and 10% Percoll containing the synaptosomes was collected. Synaptosomes were then washed with 6 ml of HBSS at 15000 x g for 15 min. The supernatant was discarded, and the pellet resuspended in 1 ml HBSS and centrifuged at 10000 X g for 10 min. The final pellet was resuspended in 100 µL of RIPA buffer (Pierce 89900) and stored at -80°C for later analysis by ELISA.

### VGLUT1 and PSD95 quantification

Once all synaptosome samples were obtained, VGLUT1 and PSD95 were measured via ELISA according to the manufacturer’s specifications (LSBio, Shirley, MA). Concentrations of VGLUT1 and PSD95 were normalized to tissue weight to account for any variances in quantity of tissue collected.

### Synapse quantification by confocal microscopy

Mice (n=12 for wild type and C3 KO; n=4–6 for wild type and C5 KO experiments) were sacrificed using an overdose of inhaled isoflurane at day 28 post-infection. After euthanasia, a peristaltic pump (Rainin Dynamax RP-1) was used to deliver ice-cold PBS (HyClone SH30256.02) followed by 4% PFA (Fisher BP531) in PBS via transcardial perfusion at a rate of 10 ml/min. Once perfused, the catheter was removed from the brain and the brain was placed in 10 ml of 4% PFA on ice. The brains were allowed to remain in PFA and returned to 4°C for a minimum of 18–24 h.

To section brains, a 3% agar base was glued to the vibratome (Leica VT 1000S) base. Fixed brains were removed from 4% PFA and rinsed 3 times in PBS. The brain was trimmed and glued to the agar base and placed in the slicing chamber with PBS to create 100 µm coronal sections. The brain was sectioned collecting the coronal slices in order and sequential slices were placed in a 24 well plate with sterile PBS. Once the plate was full it was wrapped in foil and stored at 4°C until used for immunohistochemistry.

For immunohistochemistry, each brain section was washed for 5 min in 1X PBS, then the PBS was removed and 300 μL of permeabilization buffer, 0.3% Triton (Fisher #BP151–100) in 1X PBS, was added for 3 minutes. Next, the permeabilization buffer was removed and 300 μL blocking buffer, permeabilization buffer with 5% goat serum (Vector labs #S-1000), was added for 1 h. Primary antibodies for VGLUT1 (Millipore Cat# AB5905, RRID: AB_2301751) and PSD95 (Thermo Fisher Scientific Cat# MA1–045, RRID: AB_325399) were diluted 1:500 with permeabilization buffer and 5% normal goat serum (Vector Laboratories S-1000, Newark, CA). Slices were incubated with antibodies at 4°C overnight with constant rocking.

The next day the primary antibody solution was removed followed by three washes with 1X PBS at room temperature. Then 300 μL of secondary antibody diluted to the following concentrations in permeabilization buffer with 1% NGS: 1:500 Alexa Flour 488 ms (Thermo Fisher Scientific Cat# A-11001, RRID: AB_2534069) and 1:300 Alexa Flour 647 guinea pig (Thermo Fisher Scientific Cat# A-21450, RRID: AB_141882). Slices were incubated at room temperature for 90 min followed by 3 washes for 10 min with 1X PBS. Finally, 300 μL of 1:300 DAPI (BD Biosciences Cat# 564907, RRID: AB_2869624) diluted in 1X PBS for 10 min followed by a PBS wash. Stained slices were then mounted to slides with Fluoro-Gel with Tris buffer (Electron Microscopy Sciences 17985–10, Hatfield, PA).

Confocal imaging was performed on a Zeiss LSM 700 using a 40 X 1.4 N.A oil lens. Images (8 bit, 512 x 512 pixels with pixel size of 0.21 µm and Z step of 0.20 µm) were collected in layer 5 of the somatosensory region at 100 µm from the catheter tract to limit influence of tissue damage from catheter insertion using 488, 555 and 659 nm lasers (35). Laser power and gain settings were kept constant for all experiments (35). All measurements were made within 10 µm from the surface of the section to ensure uniformity of antibody penetration (35). Layer V of the cortex was identified by density of DAPI staining.

Once acquired, confocal images were analyzed with the Puncta Analyzer program for quantification, which is an Image J 1.26 plug-in (36). A region of interest (ROI) of 25 x 25 x 2 µm was identified in layer 5 of the somatosensory cortex (35). Pre-synaptic puncta as identified by VGLUT1 staining, post-synaptic puncta as identified by PSD95 staining, and total synaptic puncta, as defined by pre- and post-synaptic marker co-localization were quantified by Puncta Analyzer and averaged over 5 sequential Z- stacks per section and then averaged over two tissue sections per animal (35, 36).

### Data analysis

Significant differences between sterile and *S. epidermidis* infected or wild type and C3 or C5 KO mice were determined using the Wilcoxon signed-rank test with Prism GraphPad (Boston, MA) at the 95% confidence interval. A *p* value of less than 0.05 was considered statistically significant.

## Results

### C1q is significantly elevated during chronic CNS catheter infection

**Figure 1:**
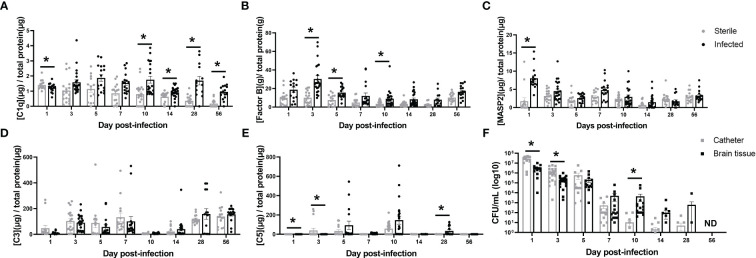
Complement component kinetics from the classical **(A)**, Alternative **(B)**, and Lectin **(C)** pathways as well as the converged pathway **(D, E)** over the course of infection in brain tissue surrounding the catheter insertion site from wild type mice implanted with sterile or *Staphylococcus epidermidis* infected catheters. Corresponding catheter and brain tissue bacterial burdens from animals with *Staphylococcus epidermidis* infected catheters **(F)**. Animals with sterile catheters had no growth from catheters or brain tissue (data not shown). *p<0.05, ND, not detected.

To explore which complement activation states persist throughout the course of CNS catheter infection, complement components from each of the three activation pathways (classical [C1q], alternative [Factor B] and lectin [MASP2]) as well the converged pathway (C3) and the terminal pathway (C5) were quantified in the brain parenchyma surrounding catheter implantation site in sterile versus infected C57BL/6 mice along with biofilm bacterial burden from the catheter and bacterial burden in the surrounding brain parenchyma (**Figure 1**). Mice receiving sterile catheters were used to compare the post-operative inflammatory response to active infection. MASP2 was significantly increased at day one post-infection, suggesting that the lectin pathway predominates at this time point (**Figure 1C**). As infection progressed, the alternative pathway was elevated as evidenced by significant increases in Factor B at days 3, 5, and 10 post-infection (**Figure 1B**). Interestingly, during mid to late infection (i.e., days 10–56), C1q levels were significantly elevated when bacterial burdens were low or below the limit of detection, suggesting a potential role for the classical pathway beyond pathogen recognition (**Figures 1A, F**). While C3 levels changed throughout the time course, levels were comparable in both sterile and infected animals (**Figure 1D**). C5 levels also fluctuated throughout the infection period with concentrations higher in sterile animals early in infection but later in the post-operative course were increased in mice with *S. epidermidis* infected catheters (**Figure 1E**). Bacterial burdens were highest during the acute post-operative period with significantly more bacteria adherent to the catheter at early intervals post-infection, reflective of biofilm growth (**Figure 1F**). As the time from infection increased, bacterial burdens progressively declined. At day 5 there were a small number of animals with undetectable bacterial burdens which then increased over time. By day 56, the infection was cleared with no recoverable organisms present on catheters or the surrounding brain tissue. Over the course of infection there appeared to be a shift from biofilm- to a parenchymal-based infection as bacterial abundance was elevated in the brain tissue compared to catheters starting at day 7 post-infection. Animals with sterile catheters had no growth from catheters or brain tissue throughout the interval examined.

### C3 and C5 do not affect synaptic abundance during *S. epidermidis* CNS catheter infection

**Figure 2:**
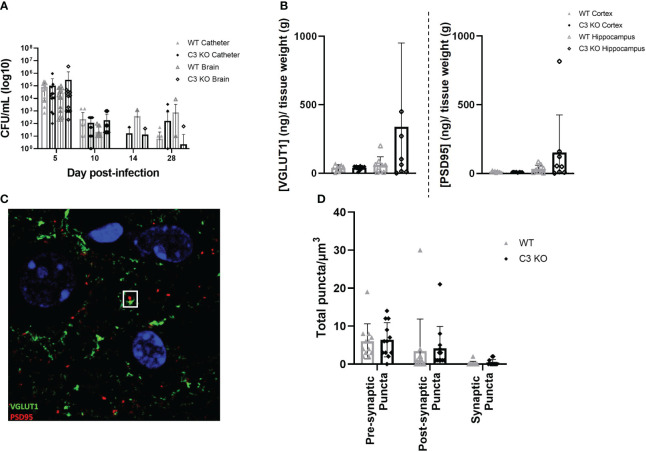
Bacterial burdens from catheters and surrounding brain tissue in wild type (WT) and C3 knockout (KO) animals implanted with *S. epidermidis* infected catheters **(A)**. Pre synaptic VGLUT1 and post synaptic PSD95 in the cortex and hippocampus of wild type and C3 KO animals implanted with *S. epidermidis* infected catheters at day 28 post-infection **(B)**. Representative Z projection from layer five of the cortex, VGLUT1 labeled in green and PSD95 labeled in red, with a synaptic punctum identified in the white square **(C)**. Pre-synaptic puncta, post-synaptic puncta and synaptic puncta quantified in layer 5 of the cortex of WT and C3 KO animals implanted with *S. epidermidis* infected catheters at day 28 post-infection **(D)**.

As C3 is an essential component of the innate immune response to infection and is the point of convergence for all complement activation pathways, we examined if the absence of C3 would alter the host response to *S. epidermidis* CNS catheter infection. Bacterial burdens on catheters and in the surrounding brain tissue were quantified in C57BL/6 mice and C3 knockout (KO) mice which are on a C57BL/6 background (**Figure 2A**). There were no statistically significant differences in bacterial abundance on catheters or in brain tissue for wild type C57BL/6 compared to C3 KO mice, indicating the absence of C3 does not have a negative impact on pathogen control. Since complement components have been implicated in synaptic pruning during various neuroinflammatory insults, we next determined whether synapse density was affected in C3 or C5 KO mice during *S. epidermidis* CNS catheter infection. First, VGLUT1 and PSD95 levels were quantified in synaptosomes recovered from the cortex surrounding the catheter insertion site as well as the adjacent hippocampus. No significant differences in either VGLUT1 or PSD95 were observed between wild type and C3 KO mice (**Figure 2B**) in either brain region. Next, confocal imaging was used to quantify pre-synaptic (VGLUT1) and post-synaptic (PSD95) puncta as well as total synaptic puncta by co-localization of VGLUT1 and PSD95 in layer V of the cortex (**Figures 2C, D**). No significant differences in number of puncta were observed between wild type and C3 KO *S. epidermidis* infected mice.

Similar experiments were performed in C5 KO and C57BL/10SnJ wild type mice to determine if C5 affected synaptic density. Like C3, C5 loss did not impact bacterial abundance, with similar numbers of bacteria on catheters and in the surrounding brain tissue of both wild type and C5 KO animals over the course of infection (**Supplementary Figure 1A**). Confocal imaging did not detect a difference in pre-, post- or total synaptic puncta in wild type compared to C5 KO *S. epidermidis* infected animals in layer V of the cortex adjacent to the catheter insertion site (**Supplementary Figure 1B**).

## Discussion

In the present study we found that the abundance of complement components varied over the post-operative course in animals with both sterile and *S. epidermidis* infected catheters. Surprisingly, some complement proteins were significantly increased at later intervals when bacterial burdens were minimal or undetectable. MASP2 was significantly increased in animals with *S. epidermidis* infected catheters at post-operative day one, suggesting that the lectin pathway may be a primary mode of complement activation at least initially. This would be consistent with mannose binding lectin recognizing *S. epidermidis* glycoproteins. MASP2 levels remained elevated in animals with infected compared to sterile catheters from days 3 through 14, indicating continued activation of the lectin pathway. During these intermediate time points, Factor B was also significantly increased in mice with infected catheters, which is consistent with our previous work, which found elevated Factor B in the CSF of rats with *S. epidermidis* CNS catheter infection (16). Elevations of these two mediators at intermediate time points suggests activation of both the alternative and lectin pathways, which is expected with the Gram-positive pathogen *S. epidermidis*. Mannose binding lectin would recognize and bind to glycoproteins on the surface of *S. epidermidis* leading to C3b deposition. Early elevations of MASP2 make it an attractive diagnostic biomarker candidate as its presence corresponds to infection and can differentiate active infection from the sterile post-operative inflammation associated with catheter insertion. The chronic footprint of Factor B may also potentially be attractive as a biomarker especially as its levels drop off with a decrease in bacteria burdens. CSF Factor B levels could potentially be tracked during treatment and used to determine individualized antibiotic durations, reducing the many potential harms of antibiotic overuse. Clinical studies are underway to further explore the utility of MASP2 as a diagnostic biomarker as well as Factor B as a monitoring biomarker.

C1q, from the classical pathway, was dramatically increased in animals with *S. epidermidis* infected catheters at late timepoints, especially days 28 and 56 post-infection. This is intriguing as bacterial burdens were undetectable on catheters or surrounding brain tissue at day 56, suggesting a role for C1q beyond pathogen opsonization and control. There is growing evidence that C1q has many non-traditional functions in the CNS, most notably its roles in synaptic pruning and modulating the migration and fate of neural stem cells (37, 38). C1q has been identified as a key player in synaptic pruning during normal development and in neuroinflammatory conditions such as Alzheimer’s Disease (17, 39). It has been well established that C1q plays an active role in normal synaptic pruning during development of the retinogeniculate system (40). Additionally, C1q induces microglial-mediated synaptic clearance in Alzheimer’s disease (39). C1q and C3a have been identified as drivers of neural stem cell migration and cell lineage selection (37, 41). Beyond these known roles, elevated C1q levels have been associated with many neuroinflammatory disease states, although there is not always a clear delineation between a detrimental or beneficial role in these conditions (38). Emerging evidence has shown that mechanisms of synaptic pruning are region- and context-dependent. The current report has clearly established elevated C1q levels during late *S. epidermidis* CNS catheter infection, which supports our prior studies demonstrating elevated C1q in the CSF of a rat model of *S. epidermidis* CNS catheter infection (16). However, more work is needed to determine whether C1q is playing a beneficial or deleterious role in these infections and the mechanism through which it is exerting its effects.

Because C3 is the point at which all three complement pathways converge and drives many of the effector functions of the cascade such as bacterial clearance and inflammatory cytokine production, we examined how C3 loss affected bacterial abundance. Interestingly, we found that the absence of C3 does not have a major effect on *S. epidermidis* growth in catheter-associated biofilm or in the surrounding brain parenchyma. This finding raises the possibility that complement inhibitors could be safely used in human shunt infection if there was a role in mitigating neurologic morbidity, since C3 and the downstream terminal pathway were not essential for controlling CNS catheter infection. This was further reinforced by our C5 KO data where there was again no statistical difference in bacterial burdens between catheters and brain tissue in wild type and C5 KO animals with *S. epidermidis* infected catheters. C3 and C5 are key factors in pathogen response, our data demonstrating that bacterial abundance was not affected suggest potential safety from a pathogen response standpoint of complement inhibition as all pathways converge on the terminal pathway. However, a thorough investigation of the underlying mechanism and potential compensatory pathways is warranted as well as the effect of C1q loss or inhibition as it appears to play a larger role in these infection than C3 and C5.

There is increasing evidence that C3 also plays a role a role in synaptic pruning (17). Therefore, we examined the possibility of C3-mediated synaptic loss during *S. epidermidis* CNS catheter infection. We assessed the levels of both pre-, VGLUT1 a pre-synaptic transporter highly expressed in the cerebral cortex and hippocampus, and post-, PSD95 a ubiquitous post synaptic protein, synaptic proteins in the cortex immediately surrounding the catheter insertion site as well as the hippocampus as this is an anatomically adjacent site and critical for long-term potentiation, and complement effects are known to be brain region-specific (42–45). After isolating synaptosomes from these two sites in wild type and C3 KO animals with *S. epidermidis* infected catheters, VGLUT1 and PSD95 were quantified. Not finding any differences between wild type and C3 KO mice in VGLUT1 or PSD95 abundance in either area, we then took a deeper look at synaptic density in the cortex immediately surrounding the catheter as this area was expected to be most highly impacted by infection and the resultant immune response based on proximity. No differences in pre-, post- or total synaptic puncta were observed in this region. Collectively, this suggests that C3 does not appear to play a large role in synaptic abundance during *S. epidermidis* CNS catheter infection.

While there is less evidence for a direct role of C5 in synaptic loss, C5, and more specifically C5a, have been shown to play important functions in the CNS. For example, the C5a-C5aR1 axis is critical for human embryonic brain neurogenesis and C5a is a potent neuroinflammatory mediator (46). As mentioned earlier, the absence of C5 did not impact bacterial growth or pre-, post- or total synaptic puncta abundance in layer V of the cortex immediately surrounding the catheter insertion site in wild type or C5 KO mice. Therefore, if synaptic loss contributes to the neurologic damage associated with CNS catheter infection, which remains an outstanding question, it is independent of either C3 or C5.

While several complement components have been implicated in important roles during neurologic development, neurodegeneration, and neuroinflammatory states, these effects appear to be context- and time-dependent. We have demonstrated that the lectin and alternative pathways are most abundant during early and mid CNS catheter infection, whereas the classical pathway becomes elevated during mid-infection and remains elevated in late infection when bacterial burdens are low or undetectable. Based on the continued elevation of C1q in response to *S. epidermidis* CNS catheter infection and the wealth of information demonstrating a significant role for C1q in neurodevelopment and neuroinflammatory states, future studies will focus on the effects of C1q in these infections.

## Data availability statement

The raw data supporting the conclusions of this article will be made available by the authors, without undue reservation.

## Ethics statement

The animal study was approved by The Institutional Animal Care and Use Committee at the University of Nebraska Medical Center approved the protocol for animal use (protocol #19-029-05) and is compliant with the National Institute of Health guidelines for the use of rodents. The study is reported in accordance with ARRIVE guidelines. The study was conducted in accordance with the local legislation and institutional requirements.

## Author contributions

MB: Formal analysis, Investigation, Writing – review & editing. LB: Conceptualization, Investigation, Writing – review & editing. AD: Conceptualization, Funding acquisition, Methodology, Writing – review & editing. TK: Conceptualization, Funding acquisition, Resources, Visualization, Writing – original draft. GS: Conceptualization, Formal analysis, Funding acquisition, Investigation, Methodology, Project administration, Supervision, Visualization, Writing – original draft, Writing – review & editing.

## Supplementary material

Supplementary Figure 1 Bacterial burdens from catheters and brain tissue in wild type (WT) and C5 knockout (KO) animals implanted with *S. epidermidis* infected catheters **(A)**. Pre-synaptic puncta, post-synaptic puncta and synaptic puncta quantified in layer 5 of the cortex of WT and C5 KO animals implanted with *S. epidermidis* infected catheters at day 28 post-infection **(B)**.
