# Supplementary figures and images for: C1q is elevated during chronic Staphylococcus epidermidis central nervous system catheter infection
Source: Front Immunol. 2024 May 31;15:1342467. doi: 10.3389/fimmu.2024.1342467 (PMC11176433; doi:10.3389/fimmu.2024.1342467)

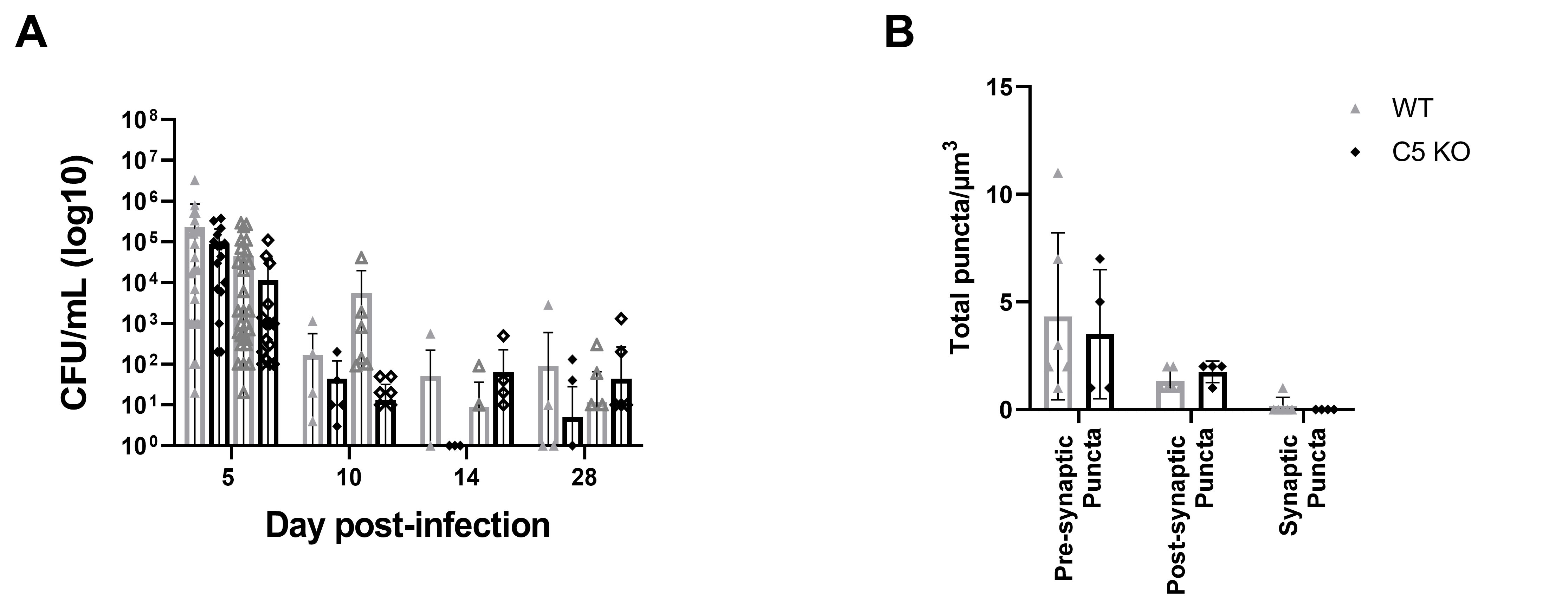

Supplement: Supplementary Figure 1 — Bacterial burdens from catheters and brain tissue in wild type (WT) and C5 knockout (KO) animals implanted with S. epidermidis infected catheters (A). Pre-synaptic puncta, post-synaptic puncta and synaptic puncta quantified in layer 5 of the cortex of WT and C5 KO animals implanted with S. epidermidis infected catheters at day 28 post-infection (B). [file Image_1.jpeg]
